# New Insights into Cervicofacial Vascular Anomalies

**DOI:** 10.3390/jcm13123515

**Published:** 2024-06-15

**Authors:** Daniela Vrinceanu, Mihai Dumitru, Andreea Marinescu, Bogdan Dorobat, Octavian Dragos Palade, Felicia Manole, Horia Muresian, Matei Popa-Cherecheanu, Cătălina Mariana Ciornei

**Affiliations:** 1 ENT Department, Carol Davila University of Medicine and Pharmacy, 050472 Bucharest, Romania; vrinceanudana@yahoo.com; 2 Radiology Department, Carol Davila University of Medicine and Pharmacy, 020021 Bucharest, Romania; andreea_marinescu2003@yahoo.com (A.M.); bdorobat@gmail.com (B.D.); 3 Surgical Department, Faculty of Medicine, University of Medicine and Pharmacy “Grigore T. Popa”, 700115 Iasi, Romania; drpalade@gmail.com; 4 ENT Department, Faculty of Medicine, University of Oradea, 410073 Oradea, Romania; manole.felicia@gmail.com; 5 Vascular Surgery Department, Bucharest University Emergency Hospital, 050098 Bucharest, Romania; cvsurg@hotmail.com; 6 Department of Cardiovascular Surgery, Carol Davila University of Medicine and Pharmacy, 011356 Bucharest, Romania; matei.cherecheanu@gmail.com; 7 Department of Physiology, Carol Davila University of Medicine and Pharmacy, 050474 Bucharest, Romania; catalina.ciornei@umfcd.ro

**Keywords:** vascular tumor, vascular malformation, imaging, angiography, embolization, surgery

## Abstract

Congenital cervicofacial vascular anomalies are extremely rare and present many difficulties in diagnosis and treatment requiring a multidisciplinary approach. Firstly, there is little consensus on this subject among head and neck specialists. There are two main types of vascular anomalies: vascular tumors and vascular malformations. Vascular malformations are also divided into malformations with slow blood flow (veins, lymphatics, capillaries or combined) and malformations with a fast blood flow (arteriovenous malformations and fistula). Vascular tumors like hemangiomas are known for their spontaneous involution with aging, while vascular malformations grow in dimensions with age. It is very important to choose the correct differential diagnosis between cervicofacial hemangiomas and vascular malformations for proper therapy management. Anamnesis and clinical exams help in raising suspicions about the real nature of a cervico-vascular anomaly. Furthermore, imaging brings in-depth details of the anomaly, ranging from ultrasound and contrast CT to MRI scanning and minimally invasive angiography. Angiography with selective embolization is rarely a curative procedure for arteriovenous malformations, being more suitable as a preliminary step before attempted surgical removal. Surgery is clearly necessary when there are aesthetic and functional deficits. Slow-flow vascular malformations present a reduced morbidity, and in cases without involution, the surgical ablation is reserved for the cases with aesthetic dysfunctions or psychological trauma. Lymphatic malformations must undergo surgical ablation when they are associated with mass effects and compression of great vessels or aerial viscera. The prognosis after surgical removal is good, with a low rate of recurrence or morbidity. Fast-flow vascular malformations require a combined approach, with embolization and excision in the next 48 h for safety reasons. Removal may be followed by reconstructive surgery depending on the location and dimensions of the malformation, with a possible secondary recovery of the normal microscopic vessels. Some of the masses may hinder the normal airflow and swallowing. Pathology is the gold standard for confirming the clinical and imaging diagnosis.

## 1. Introduction

Cervicofacial vascular anomalies represent a complex entity in the field of vascular pathology, presenting significant challenges in diagnostic and therapeutic management. These anomalies represent malformities and lesions of the vascular system affecting the cervical and facial region, with wide variability in their clinical manifestations. Considering the impact on patients’ quality of life through aesthetic and functional aspects, the study of these lesions becomes imperative for specialists dealing with head and neck pathology. The complex anatomy of the cervicofacial region adds additional difficulty to the understanding of vascular malformations and tumors, considering the richness of the vascularization of these anatomical regions, with numerous possibilities for the development of collateral blood circulation [1].

Cervicofacial vascular anomalies represent a chapter of pathology that requires an interdisciplinary approach, starting from the standardization of terminology, classification and continuing with diagnostic and therapeutic management. Clinical cases and case series are reported in the literature, overall experience is limited, and most cases are treated with a personalized therapeutic approach. Imaging and clinical diagnosis must be very accurate to impose the appropriate therapeutic approach, which, in most cases, varies between pharmacological approach, endovascular therapy with selective embolization, surgery and “wait-and-see”. According to the International Society for the Study of Vascular Anomalies (ISSVA), vascular anomalies are classified into vascular tumors and vascular malformations [2].

Vascular tumors are mainly represented by hemangiomas. They are found in young children and, most frequently, are approached by conservative treatment (“wait-and-see”), since most of them regress spontaneously. Hemangiomas appear shortly after birth or, in rare cases, at birth, with a rapidly proliferative phase during the first 12 months of life. They then stabilize and slowly involute, with complete regression in half of cases by the age of 5 and in three quarters of cases by the age of 7 [3]. From a histological point of view, they are characterized by endothelial hyperplasia and an increased number of mast cells during the proliferative phase, while in the involutional phase, the histological appearance reveals fibrofatty replacement, diminished cellularity and a normal number of mast cells. Surgery is indicated for cases with aesthetic and functional alterations (bleeding, ulceration, pain and cervicofacial functional disorders). Head and neck hemangiomas with compression on the aerodigestive structures or labial hemangiomas causing eating and speech disorders require a therapeutic intervention [4].

Vascular malformations are, in turn, divided into slow-flow (low-flow) malformations and fast-flow (high-flow) malformations. Slow-flow malformations are venous, lymphatic, capillary, or combinations thereof, while fast-flow malformations are arteriovenous malformations and arteriovenous fistulas. Vascular malformations are always present at birth and grow progressively, become apparent most frequently in adulthood and do not resolve spontaneously. These are not tumors but abnormal clumps of vessels, histologically showing non-proliferative flat endothelium and a normal number of mast cells. Resection is usually necessary, especially for arteriovenous malformations. Also, selective preoperative embolization is increasingly used for this category of arteriovenous malformation [5].

The objectives of this review are to provide the most relevant information regarding the classification and the current diagnostic and therapeutic approach to cervicofacial vascular anomalies, based on the literature currently available. We queried PubMed for manuscripts on the subject using the following research terms: vascular malformation head and neck. We applied the following filters: Free full text, Case Reports, Clinical Study, Comparative Study, Multicenter Study, Review, Systematic Review, Humans, English, Adolescent: 13–18 years, Adult: 19+ years, Exclude preprints. The final result of the research returned 61 articles published since 2004 that are included in the present review. The search algorithm used was Search: vascular malformations head and neck Filters: Free full text, Case Reports, Clinical Study, Comparative Study, Multicenter Study, Review, Systematic Review, Humans, English, Adolescent: 13–18 years, Adult: 19+ years, Exclude preprints, from 2004–2024 ((“vascular malformations”[MeSH Terms] OR (“vascular”[All Fields] AND “malformations”[All Fields]) OR “vascular malformations”[All Fields]) AND (“head neck”[Journal] OR “head and neck”[All Fields])) AND ((ffrft[Filter]) AND (excludepreprints[Filter]) AND (casereports[Filter] OR clinicalstudy[Filter] OR comparativestudy[Filter] OR multicenterstudy[Filter] OR review[Filter] OR systematicreview[Filter]) AND (humans[Filter]) AND (english[Filter]) AND (adolescent[Filter] OR alladult[Filter]) AND (2004:2024[pdat])).

Due to the fact that multiple classifications have been published worldwide, we structured our manuscript according to the classification proposed by Kunimoto et al. [6]: simple vascular malformations (benign-capillary malformations; locally aggressive or borderline lymphatic malformations, venous malformations; high-flow malignant arteriovenous malformations, arteriovenous fistula); combined vascular malformations (defined as two or more vascular malformations found in one lesion and that can be locally aggressive or borderline); vascular malformations of major named vessels (abnormalities in the origin/course/number of major blood vessels that have anatomical names and are locally aggressive or borderline); vascular malformations associated with other anomalies (syndromes in which vascular malformations are complicated by symptoms other than vascular anomalies and are locally aggressive or borderline). Regarding the treatment used for managing various types of vascular malformations, we followed the clinical practice guidelines by Mimura et al. [7].

## 2. Cervicofacial Vascular Tumors

Vascular tumors are, in turn, divided into benign, borderline, and malignant, reflecting the diversity of the clinical and histological expression of this type of vascular anomaly. The most well-known representations of vascular tumors are hemangiomas. They are characterized by endothelial cell proliferation and angiogenesis. Hemangiomas are the most common tumors of childhood, affecting around 3% of the Asian, African, and Caucasian pediatric population, with a female predominance. Among affected children, 20% have multiple lesions, and the rest have solitary lesions [8].

### 2.1. Clinical Data

Hemangiomas of the head and neck are the most numerous (60–70% of cases), followed by those of the trunk (a quarter of cases) and those of the limbs (5% of cases). Infantile hemangiomas (IHs) are the most common (90%), while congenital hemangiomas account for less than 2%. Infantile hemangiomas tend to grow rapidly in the first months after birth, with spontaneous regression in early childhood. Congenital hemangiomas, unlike infantile hemangiomas, are present from birth and are subdivided into rapidly regressing hemangiomas, partially regressing hemangiomas, and non-regressing hemangiomas [9]. Moreover, the differentiation between congenital and infantile hemangioma is important also for choosing an effective treatment strategy; for example, Timolol is ineffective in congenital hemangiomas and might even increase the likelihood of adverse effects [10].

**Figure 1:**
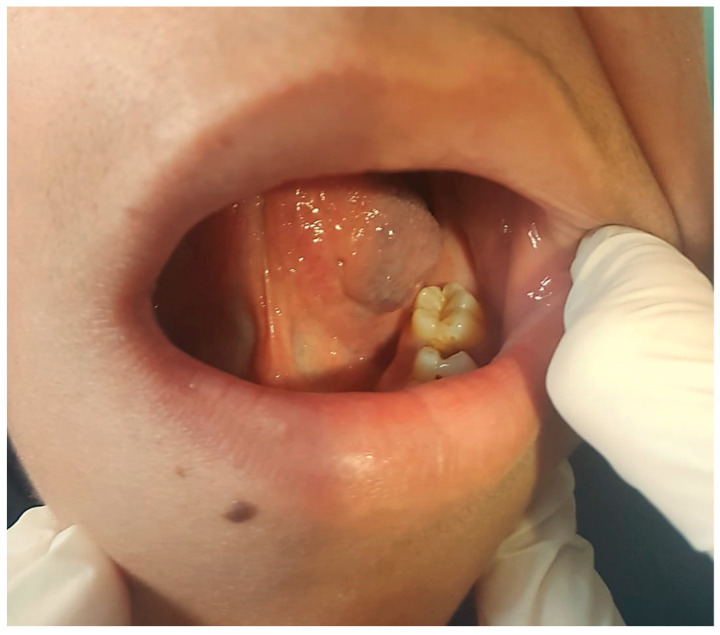
Infantile hemangioma on the lateral left aspect of the tongue—clinical aspect.

Almost all cervicofacial hemangiomas are easily diagnosed based on clinical diagnosis alone. They are represented by superficial, reddish, bumpy, strawberry-like (strawberry-like or raspberry-like) lesions. They can be skin lesions, subcutaneous, in the deep dermis or in the parenchyma of organs, apparently with a normal overlying skin or as bluish or violet masses, prone to confusion with venous malformations. Clinical examination and history are essential for proper diagnosis. On palpation, the lesion feels firm, incompressible, without thrill (palpatory counterpart of murmur), becoming less firm and undergoing a process of discoloration with involution. Signs and symptoms at presentation are varied depending on tumor location (Figure 1). The vascular tumor appears as a cervicofacial swelling or at the level of the oral cavity, with color changes at the level of the skin or mucous membranes [11]. Imaging is rarely employed for the diagnosis of infantile/congenital hemangiomas, but US serves as the primary choice for uncertain clinical diagnoses (deep lesions, objectively difficult-to-assess lesions, and for evaluating the extent of a deep lesion or searching for associated anomalies) or for monitoring the response to medical therapy. In children with segmental infantile cutaneous hemangiomas, magnetic resonance imaging (MRI) is the reference method to highlight deep localizations and/or any associated malformities, as seen in syndromic forms. Histological examination provides a valuable adjunct to clinical and imaging investigations to differentiate benign vascular lesions from malignant ones [12].

### 2.2. Diagnosis

The elements that bring the patient to the doctor are represented by bleeding, pain, swallowing or speech disorders, as well as elements related to the unaesthetic appearance that can be associated with psychosocial trauma. A separate chapter is represented by lingual and pelvilingual hemangiomas which, depending on their size and extent, can cause swallowing, mastication, and speech disorders. Trauma by self-biting or secondary to crunchy foods in the case of hemangiomas of the oral cavity and pharynx can represent irritating spines for bleeding or the increase in size of the lesions [13].

**Figure 2:**
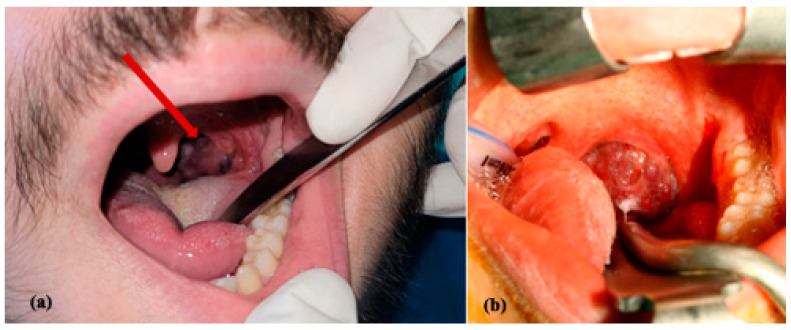
Clinical aspect (**a**) and during surgery (**b**) of a patient with congenital vascular tumor of the left wall of the pharynx associated with swallowing dysfunction.

**Figure 3:**
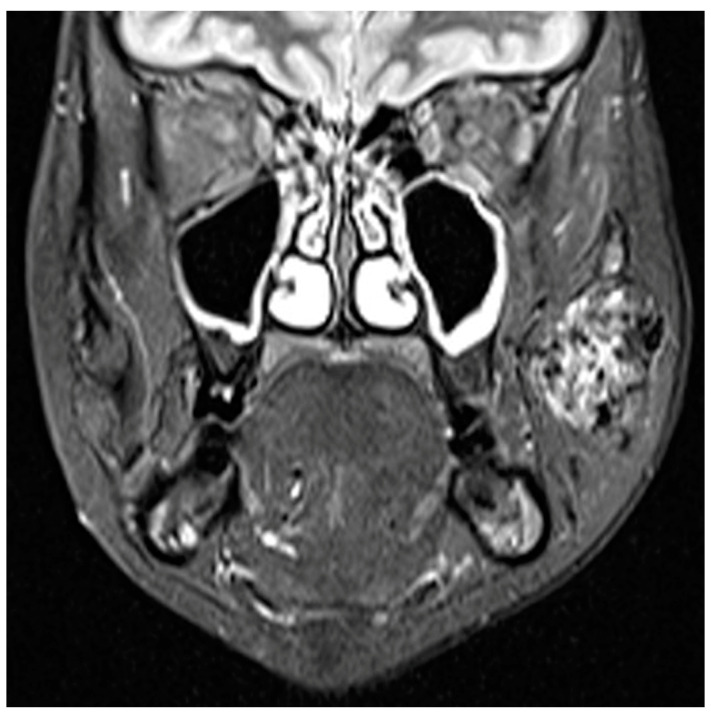
MRI imaging of a vascular tumor of the left side of the face with T2 hypersignal suggesting an infantile hemangioma.

**Figure 4:**
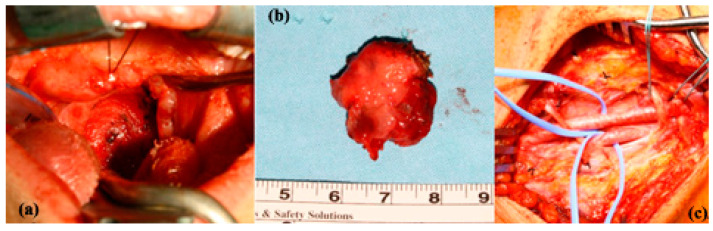
Surgical aspect of a congenital vascular tumor on the left lateral wall of the pharynx with radical ablation of the lesion (**a**,**b**), after performing exploratory left neck dissection in order to prepare the neck vessels for rapid access in case of major bleeding requiring emergency blood vessel ligation (**c**).

**Figure 5:**
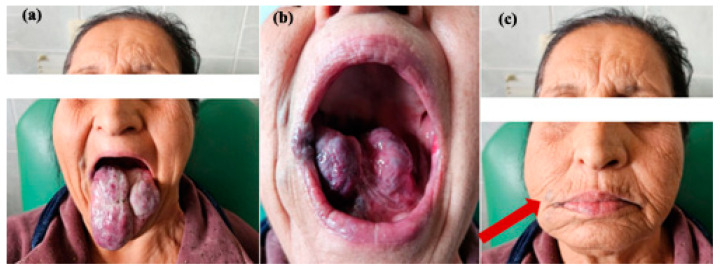
Clinical aspect of a giant right lingual venous malformation and adjoining venous malformation from the right facial vein (red arrow), without indication for surgery, (**a**) aspect of the tongue, (**b**) aspect of the tongue floor, (**c**) aspect of the corner of the mouth.

In the case of this type of vascular tumor, careful endoscopic endocavitary investigation and MRI with contrast are indicated. Surgical ablation can permanently resolve the lesion, with minimal functional sequelae (Figure 2, Figure 3 and Figure 4). There are also lesions that, by size and extension, as well as by association with other vascular malformations of the venous type, contraindicate surgical intervention (Figure 5). The indication for treatment is limited to infantile hemangiomas (IHs) at risk of life-threatening complications (high-output cardiac failure or obstruction/compression of the airways), at risk of functional impairment (vision, nutrition, hearing, and manual abilities), significant and/or permanent aesthetic damage, and ulcerated IHs that do not respond to standard topical treatments. Medical therapy with oral propranolol at a dose of 2–3 mg/kg/day is the first-line treatment, as it has demonstrated greater efficacy and safety compared to oral corticosteroids [14].

Vascular tumors include other entities besides hemangiomas that represent the benign variant. Locally aggressive (Kaposiform hemangioendothelioma, retiform hemangioendothelioma, papillary intralymphatic angioendothelioma, composite hemangioendothelioma), and malignant vascular tumors (angiosarcoma, epithelioid hemangioendothelioma, Kaposi sarcoma) are described [15].

**Figure 6:**
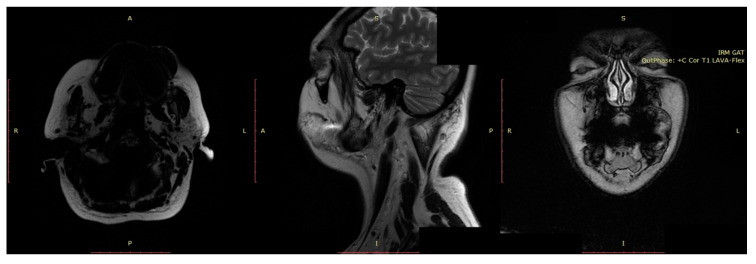
MRI of a patient with an AVM at the level of the upper lip and left aspect of the face, R—right, L—left, A—anterior, P—posterior, S—superior, I—inferior.

At ultrasound with color Doppler, an echogenic mass with a fast flow pattern is observed. CT scans with contrast media contribute to the imaging evaluation of these tumors. Contrast-enhanced magnetic resonance imaging provides the most information regarding the extent of the hemangioma. In particular, for the cervicofacial region, it is very important to evaluate other possible associated vascular injuries, as well as compression on the upper aerodigestive viscera [16]. In the T1 sequence, hemangiomas appear hyposignal to muscle, with intense hypersignal with flow-voids in post-contrast acquisitions, also known as “bag of worms” (Figure 6).

Cervical and cerebral MRI examination is mandatory for the complete evaluation of the extension of a cervicofacial hemangioma and the association with other cervicofacial and cerebral vascular abnormalities. This imaging evaluation associated with the clinical examination, which describes the functional and aesthetic impact of these vascular tumors, allows the adoption of a therapeutic strategy adapted to each individual case. Angiography is not routinely indicated in the imaging exploration of hemangiomas, and even less selective embolization, since in the case of these lesions, there is no precise arterial source to feed the vascular tumor (feeder). Exploratory angiography can be performed in the case of large hemangiomas, and selective embolization only in case of bleeding that does not stop with other usual hemostasis maneuvers [17].

### 2.3. Therapy

Therapeutic strategies for hemangiomas include drug therapy (beta blockers, corticosteroids), sclerosing agents (intratumoral steroid injections, pinyangmycin, interferon—which are now considered somewhat outdated), LASER, radiotherapy, and cryosurgery. As mentioned, most cases should be approached with a wait-and-see strategy, given the tendency for spontaneous involution. Surgery is reserved for cases with aesthetic and functional deficits (chronic unaesthetic appearance, pain symptoms, dysphagia, eating and speech disorders, as well as the need for differential diagnosis) [18]. Medical therapy with oral propranolol is the first-line treatment in these cases. Surgery is reserved for infantile hemangiomas that are resistant to medical therapy or show an unsatisfactory response, or in cases where medical therapy is contraindicated. Another indication for early surgery is the prevention of growth disturbances in the involved structures to ensure their proper development. Additionally, surgery is indicated for the treatment of sequelae resulting from hemangiomas (e.g., ulceration after rapid involution) [19].

**Figure 7:**
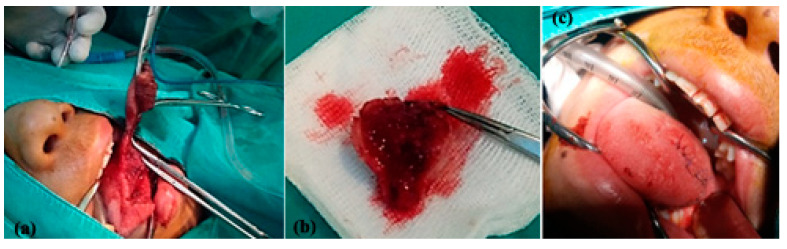
Surgical removal of a left tongue hemangioma in a young boy, (**a**)—surgical aspect, (**b**)—resection piece, (**c**)—reconstruction stage.

**Figure 8:**
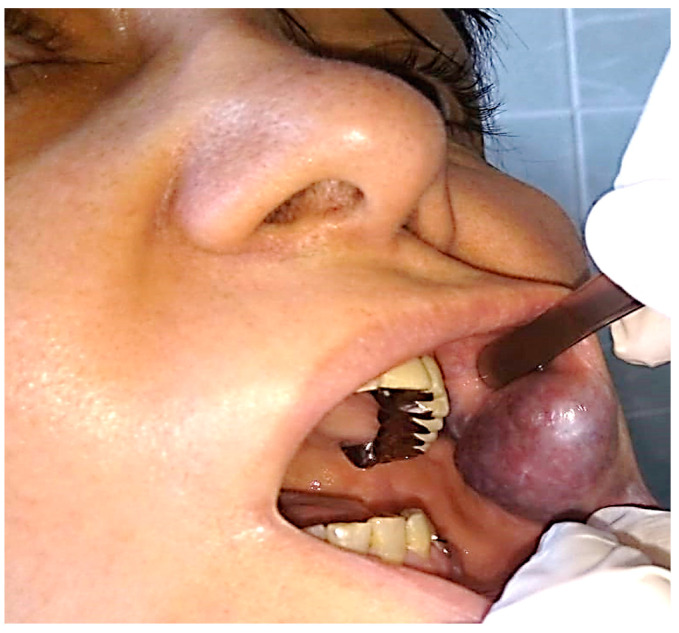
Hemangioma at the level of the left cheek after local trauma due to improper dental restoration.

We must remember that surgery is not the treatment of first choice; in the case of children, it is considered rather preventive and requires the informed consent of the parents (Figure 7). Usually in adults, cervicofacial hemangiomas are indicated for surgery because the patient knows about his lesion from childhood, may have had minimally invasive non-surgical treatments, and comes to the doctor because of new symptoms and signs that have appeared (e.g., hemangioma increased in volume, bleeding, superinfection after a local trauma) (Figure 8).

Cases in which the surgical indication is formulated must be carefully investigated by imaging, mandatorily by computer tomography (CT) and MRI, as mentioned before, which evaluate the extension of the hemangioma to the cervicofacial structures, possible intracranial extensions, or other associated anomalies (for example, PHACE syndrome in which extensive facial hemangiomas are correlated with ocular, cardiac, vascular, and central nervous system malformations) [20].

Surgical ablation, especially for deep lesions, can differentiate a malignant lesion from a non-involuting or partially involuted hemangioma. Histological examination of surgical specimens reveals endothelial cells with multilaminated basilar membranes and numerous mast cells. Immunohistochemistry reveals GLUT-1 as a routine marker to adequately differentiate GLUT-1-positive hemangiomas from vascular malformations [21].

## 3. Vascular Malformations

Vascular malformations consist of angiogenetic and vasculogenic dysplasia, are always present at birth, never regress spontaneously, and may increase in size over time. They can be asymptomatic for a long time. As they grow, they can cause pain and functional deficits. Practically, half of the vascular malformations are found in the oral and maxillofacial region. Due to this location, the clinical onset of cervicofacial vascular malformations can be through fatal hemorrhagic accidents following trauma, dental extractions, or surgical interventions. Vascular malformations can be composed of a single type of blood vessel, combined vascular components, and vascular malformations with additional non-vascular abnormalities, classified as vascular malformations or combined vascular malformations [22].

### 3.1. Venous Malformations

Like hemangiomas, the diagnosis of venous malformations is relatively easy by clinical examination. Venous malformations (VMs) are the most common symptomatic vascular abnormalities, presenting in the older child or young adult with bluish skin color, local swelling, and pain. From a clinical point of view, they are soft compressible masses, with bluish overlying membranes or mucous membranes, without thrill or pulsation. The skin or mucous membrane may have bluish closed areas that may indicate phleboliths after recurrent thrombophlebitis. They can be located cutaneously, subcutaneously, under the muscle fascia or at bone level [23].

#### 3.1.1. Clinical Data

A special category is represented by venous vascular malformations, located in the mucosa and submucosa, and which must be differentiated from hemangiomas. Cervicofacial venous malformations located especially in the facial vein territory can range from discrete lesions to bulky vascular malformations that cause significant facial asymmetry and progressive functional impairment. There is no description of a predilection for this type of malformation according to sex [24].

#### 3.1.2. Diagnosis

Imaging is necessary for indeterminate lesions and to appreciate the extent of lesions and associated abnormalities. Certain bulky vascular malformations are associated with developmental abnormalities of the pericranial and intracranial venous sinuses [25].

On standard radiological examinations and CT scans, venous malformations typically appear as soft tissue tumors containing phleboliths. CT scan examination with contrast is useful in assessing bone invasion and to assess the potential extra-intracranial communication [26].

The flow pattern of VMs on ultrasound is slow-flow or no-flow in case of thrombosis. They appear as hypoechogenic clusters of compressible, dysplastic veins in every tissue layer. The Puig classification divides these venous malformations after venous drainage into venous malformations type I, without venous drainage, venous malformations type II, with drainage into normal veins, venous malformations type III, with drainage into dysplastic veins, and venous malformations type IV, with drainage into a network of dilated veins [27].

MRI plays an important role in diagnosis, but also in monitoring the evolution. In T2-weighted sequences, venous malformations show an intense hypersignal, while in precontrast T1-weighted sequences, they show isosignal with muscle, having hypersignal in post-contrast T1-weighted sequences. Gadolinium-type contrast loading is patchier and more central, compared to lymphatic malformations that have at most minimal peripheral loading [28].

Angiography is not a routine investigation, having an invasive character, but it brings useful information in voluminous cervicofacial malformations. In venous malformations, angiography is not indicated. In cases of suspected arteriovenous malformation, it is advisable to perform CT angiography [29].

#### 3.1.3. Therapy

Regarding the therapeutic strategy for venous malformations, in most locations, they can be treated conservatively using compression therapy for local swelling and pain and aspirin for thrombosis prophylaxis. In contrast, cervicofacial VMs have significant aesthetic impact and sometimes require active treatment. This type of treatment may include sclerotherapy, embolization, surgical resection, or a combination of these techniques. Scleroembolization of venous malformations with ethanol or foam with polidocanol does not result in bleeding, and swelling is transient, regressing with appropriate medical therapy. It is an effective treatment for closing venous gaps, yielding excellent results. Sequential and serial interventions over time are often required for optimal outcomes [30].

Venous malformations do not involute with age and grow progressively with the patient, also acquiring nodular aspects. Early surgical intervention is recommended, mentioning here the advantage of the psychosocial component [31].

In our experience, complete surgical excision is the treatment of choice for small and moderate-sized malformations, given the low morbidity and low recurrence rate.

We can say that special mention is deserved for the venous malformations located at the level of the lips, which frequently require serial surgical interventions, with a modest aesthetic and functional result. Zide et al. recommend the following strategies for an optimal result after surgery: for upper lip lesions—minimal over-correction of deformities (about 10%), adjustment of affected side using the contra lateral side, with correction of vermillion displacement as a final stage, with the expectation of serial corrections; for lower lip lesions—over-correction (about 20%) with subsequent serial interventions for vermillion [32]. Given that the lips are a region of high aesthetic impact, serial surgical interventions with the potential for scar formation and facial deformation are not acceptable. Therefore, excellent results can be achieved by first reducing the volume of the malformation through scleroembolization, followed by a subsequent treatment involving reduction and/or reconstructive plastic surgery [33].

Complex cervicofacial malformations can be solved by therapeutic combinations such as preoperative embolization and resection, being associated, if necessary, with plastic reconstruction methods such as skin grafts, local flaps or free flaps. Multiple procedures and serial surgeries are associated with large or multifocal vascular malformations that have a high risk of recurrence, so the surgical outcome is generally modest. Jackson IT et al. approached compartmentalization of massive vascular malformations of the head and neck using nonabsorbable sutures, followed by sclerosing injections into each compartment [34].

### 3.2. Lymphatic Malformations

Lymphatic malformations (LMs) are rarer than venous malformations. They present as local swellings, with a reddish-brown coloration of the skin. About 70% of lymphatic malformations occur in the head and neck, while 25% of lymphatic malformations are located in the trunk and extremities, and only 5% in parenchymal organs. Lymphangiomas are considered to be congenital malformations of lymphatic vessels present at birth, with 80–90% of them detected within the first two years of life [35].

#### 3.2.1. Clinical Data

About 75% of cases occur in the skin and subcutaneous tissues of the head and neck region. The submandibular region and the parotid gland are the most common locations. Most lymphangiomas are benign, soft, slow-growing tumors. Sometimes, when they become bulky, they can compress the vital structures of the head and neck and cause complications. Although more than 90% of lymphangiomas are congenital, subsequent occurrence may be the consequence of trauma, infection, neoplasm, or iatrogenic injury [36].

#### 3.2.2. Diagnosis

Histologically, lymphangiomas consist of numerous and dilated lymphatic vessels embedded in a loose fibrovascular stroma. Lymphangiomas are classified into microcystic, macrocystic, and cystic hygromas based on the size of the lymphatic cavities they contain. Capillary or microcystic lymphangiomas are composed of small, thin-walled, capillary-sized lymphatic vessels and are characteristically located in the epidermis [37].

Cavernous or macrocystic lymphangiomas are the most common and are composed of dilated lymphatic channels, characteristically invading the surrounding tissues. Cystic lymphangiomas or cystic hygromas are large macrocystic lymphangiomas filled with straw-yellow, protein-rich fluid. Microcystic lymphangiomas are composed of cysts, each measuring less than 2 cm^3^ in volume; macrocystic lymphangiomas contain cysts measuring more than 2 cm^3^ in volume; and mixed lymphangiomas contain both microcystic and macrocystic components [38].

Submucosal cystic lymphangiomas at head and neck level are very rare and must be differentiated from other vascular tumors, but also from broad types of tumors or chronic inflammatory pathology (e.g., tuberculosis) [39].

#### 3.2.3. Therapy

Lymphatic malformations rarely resolve spontaneously, and surgical excision is the most common therapeutic strategy. Other therapeutic modalities have not been shown to be as effective (e.g., aspiration puncture). In the pre-operative evaluation, their characteristic development in the form of pseudocystic, multiloculated, locoregionally extensive tumors, which deform the region and insinuate themselves between the cervical vasculo-nervous elements, must be taken into account [40]. Surgery is reserved for microcystic forms, while sclerotherapy should preferably be reserved for macrocystic lymphatic malformations, using ethanol or bleomycin as embolizing agents, especially in the head and neck region. This recommendation is supported by the difficulty in achieving effective sclerotherapy in microcystic forms and their higher propensity for progression. The likelihood of achieving radical excision in microcystic forms is generally very low, and the risk of recurrence is high. Surgery, either associated with or followed by sclerotherapy with bleomycin, is a recently utilized technique aimed at reducing the risk of recurrence [41].

Radical surgery is the only solution that can prevent recurrences, but surgical intervention is hampered by the difficulty of dissection of the noble elements of the neck such as the large cervical vessels, the primitive internal and external carotid artery, the internal jugular vein, the vagus nerve, the accessory nerve, the hypoglossal nerve and, especially, the facial nerve. Prevention of recurrences is ensured by radical ablation of all cystic lymphangioma extensions [42].

### 3.3. Arteriovenous Malformations

Arteriovenous malformations (AVMs) are rare vascular anomalies, but with numerous diagnostic and treatment problems. These are abnormal connections between arteries and veins, from which capillaries are missing, taking on the appearance of a vascular nest. In these vascular clots, the blood flows with pressure from the arteries directly into the veins, leading over time to the dilation of the veins and to the thickening of their walls, resembling the so-called arterialization of the veins [43].

#### 3.3.1. Clinical Data

There is a Schobinger classification of AVMs that is useful in the clinical evaluation of these vascular anomalies and in stratifying the degree of risk in order to establish the therapeutic protocol. From this point of view, four stages are distinguished: stage I—clinically inactive AVM, local hyperthermia of the skin; stage II—increase in arteriovenous shunt, presence of pulsations and murmur; stage III—destructive AVM, with ulceration, bleeding and pain; stage IV—decompensated AVM, with heart failure. AVMs are fast-flow vascular abnormalities due to blood circulation at their level with arterial pressure. They increase in size with age and, in this way, it becomes more and more difficult to locate the position of the arteriovenous connections, the so-called nidus of the AVM [44].

**Figure 9:**
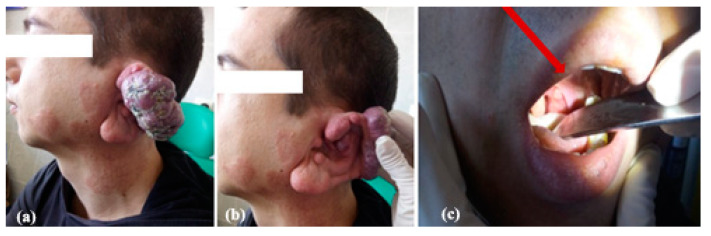
Clinical aspect of an AVM of the left ear, with wide origin at the level of the helix (**a**), association of cervicofacial capillary malformations (**b**) and venous malformations of the right tonsil (**c**, red arrow).

From a clinical point of view, AVMs can be identified at the cervicofacial level from childhood and can increase significantly in the context of trauma, pregnancy, puberty, infection or even iatrogenic trauma (biopsy, proxima feeder ligature, subtotal excision). A quarter of AVMs bleed by age 15, while most become symptomatic by age 50 (Figure 9).

On clinical examination, the overlying integument or overlying mucosa in the case of AVMs of the oral cavity and oropharynx appear normal, and beneath them a pulsatile mass with increased thrill, heat, and redness is identified. Other possible clinical signs and symptoms are pain, hemorrhage, and heart failure. Arteriovenous malformations often remain undiagnosed until dramatic bleeding from dental treatment. It is very important to examine not only the cervicofacial region, but also the nose, the oral cavity, and the pharynx by direct otorhinolaryngology examination and by endoscopy to highlight the existence of associated endopharyngeal lesions that we will have to take into account during orotracheal intubation, when choosing surgery as a therapeutic strategy [45].

#### 3.3.2. Diagnosis

Diagnostic imaging becomes extremely necessary to provide information regarding the location, extension, composition, and diameter of the feeding and draining vessels of AVMs. Color Doppler ultrasound provides a global picture of arteriovenous shunts that are frequently associated with nidal venous or arterial aneurysms by secondary degeneration of dysplastic venous walls. MRI is mandatory for complete evaluation, with identification of all elements of AVM. On MRI, the anomaly is characterized by enlarged vessels, with dilated feeding and draining vessels. There is no soft tissue mass [46].

Abnormal arteriovenous shunts are easily recognized as punctate, linear signal gaps or as hyperintensities. Flow dynamics, feeding arteries and draining veins, as well as nidus localization are best highlighted on dynamic contrast-enhanced MR angiography and post-contrast T1-weighted isovolumetric gradient echosequences. On T2-weighted imaging, AVMs appear as hypointense, tubular or nodular flow-voids. In addition to these changes, it should be noted that AVMs are never accompanied by a circumscribed, well-defined mass compared to VMs and LMs. Bone involvement in AVMs is best visualized on post-contrast T1-weighted images with intensive contrast capture of intraosseous vessels [47].

**Figure 10:**
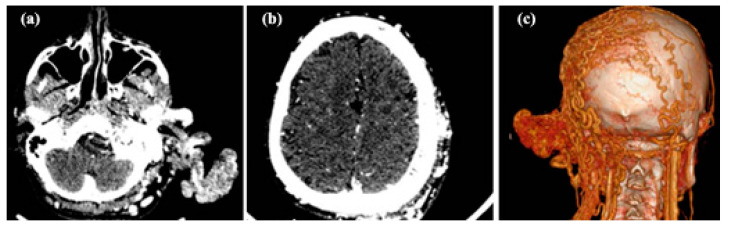
Neck CT scan (**a**) and cerebral CT scan (**b**) with contrast media and CT angiography revealing the main source of the vascular malformation from the left occipital artery and left pericrania extension (**c**).

Low-voltage CT angiography is also cited as allowing complete coverage of bulky AVMs and with easier identification of the nidus and accessibility for selective embolization (Figure 10).

**Figure 11:**
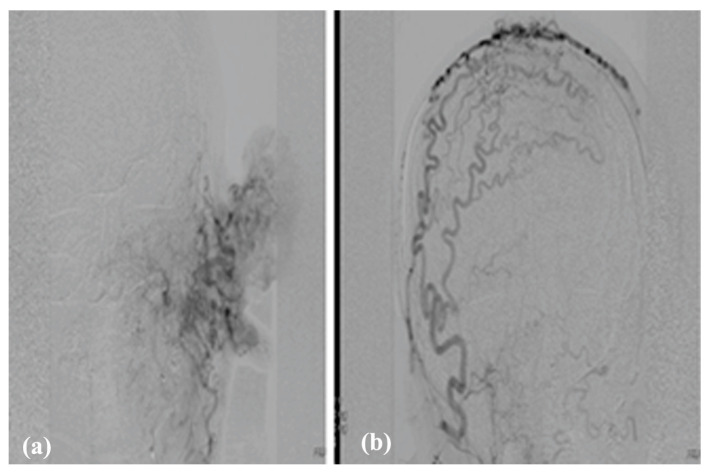
Angiography of bilateral carotid arteries of the same case with left AVM of the ear, prior to (**a**) and after (**b**) the embolization with PVA 500–700 micron particles.

Also, bilateral carotid angiography is extremely useful in highlighting the malformation and arteriovenous shunt and represents the preliminary time for embolization of the malformation with closure of the shunt. Angiography reveals high blood flow shunt within the lesion and multiple feeders in cervicofacial AVMs (Figure 11).

Feeders are usually from the branches of the external carotid artery (superior thyroid artery, facial artery, lingual artery, ascending pharyngeal artery, posterior auricular, occipital), less often from the thyrocervical trunk. Arterial and venous phases are present on the same angiographic image if there is a significant shunt. Microshunts that do not appear on angiography open after ligation of the main feeding vessels, incomplete embolization, or subtotal surgical resection, leading to clinical recurrence, with the recurrence of a malformative vascular lesion, often larger than the first lesion. Therefore, it should be mentioned that the embolization of arteriovenous malformations or arteriovenous fistulas is curative for cerebral AVMs, not being followed by malformation recurrence, since the cerebral circulation is mostly of terminal type [48].

#### 3.3.3. Therapy

**Figure 12:**
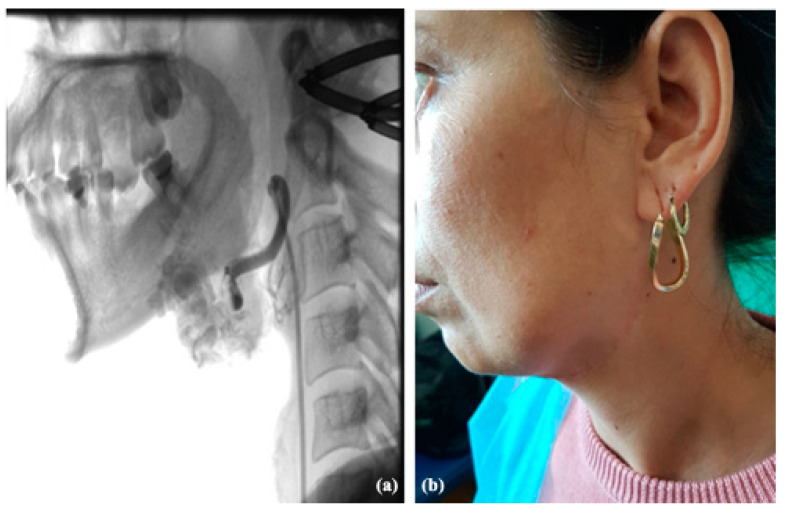
Cerebral angiography (**a**) revealing a left submandibular AVM with source from the left facial artery and post-surgical clinical aspect (**b**) and conservation of the marginal branch of the left facial nerve during the dissection of the malformation.

Regarding cervicofacial AVMs, one should take into account the numerous anastomoses between the internal and external, right and left carotid systems, which make the endovascular therapy of the malformation not work as a single therapeutic procedure, but as a preliminary time for the surgical intervention that consists of ablation malformation, with selective interruption of vascular afferents and efferents, when this is possible (Figure 12).

There are two forms of embolization: primary and preoperative. Primary embolization aims to deliver an ablative embolic agent to the nidus of AVMs to achieve endothelial destruction. This type of embolization may play a role in patients who are not ready for mutilating surgery (e.g., hemimandibulectomy for body or mandibular ramus AVMs) [49].

In preoperative embolization, the most commonly used embolization particles are polyvinyl alcohol foam (PVA) or acrylic microspheres to block the flow in the nidus, achieving preoperative devascularization, minimizing preoperative hemorrhage. Particles have different sizes, and it is important to choose the right size, because excessively large particles cause suboptimal preoperative devascularization, being equivalent to proximal ligation, while undersized particles do not serve the occlusive role and can pass into the venous circulation, causing thrombotic accidents, including pulmonary thromboembolism [50].

Certain bulky AVMs have feeders from the internal carotid artery system, and in these situations, embolization is not possible, given possible neurologic consequences. In these cases, patients must be informed about the high risk of surgical resection; the technique of compartmentalizing the malformation in case of massive bleeding, and in extreme cases, even cardiopulmonary bypass, can be taken into account [51].

Alone, endovascular therapy with selective embolization of the arterial afferents and the nidus of the malformation is followed at variable time intervals by the reappearance of the malformation, sometimes by impressive bleeding, precisely due to the opening with an aberrant component of the cervicofacial collateral circulation. However, carotid angiography remains a mandatory exploration necessary in the treatment of AVMs. This is most commonly performed transarterially (in the femoral artery, most frequently), but it can also be performed transvenously and sometimes even percutaneously, with direct access to the AVM [52].

The likelihood of definitive success in surgical intervention is linked to the radicality of the surgical excision. Partial removal, especially in pediatric or adolescent cases, may lead to a worsening of the clinical situation. Endovascular embolization is considered the first-line therapeutic approach for extensive or surgically inaccessible AVMs. The percutaneous route allows reaching the nidus directly, reducing the risks of bleeding and ulceration in high-flow malformations. Cyanoacrylate is one of the adhesive embolizing agents with a lower risk of distal embolism, ensuring instant closure of fistulas [53].

**Figure 13:**
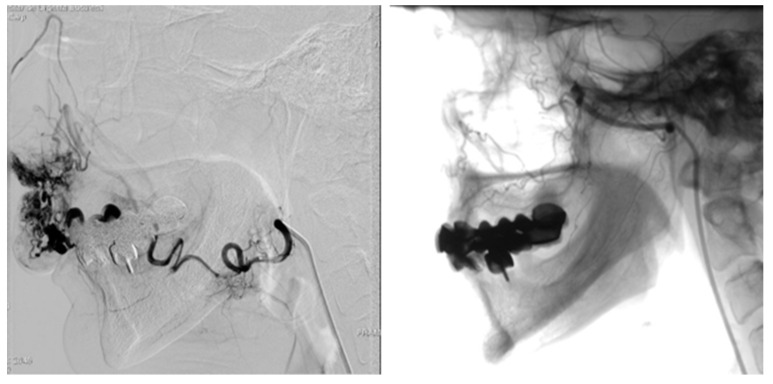
Angiography of the AVM from the level of the superior lip with main source from the right superior labial artery before and after the selective embolization.

Angiography highlights flow dynamics, feeding afferents, draining veins and malformation nidus. Embolization can be performed in the same session as angiography, if the surgical indication is established (Figure 13).

Compared to hemangiomas and venous malformations, AVMs are rare in the head and neck, and surgeon experience is limited. Kohout et al. recommend embolization with resection for stages I and II in order to prevent lesion progression, while a rapidly growing and painful lesion benefits from early intervention, preventing progression and the risk of major hemorrhage [54].

Embolization alone, subtotal resection or proximal ligation of feeders are not effective procedures due to the opening of new collaterals at the level of cervicofacial malformation, taking into account the numerous anastomoses in the carotid system. In the surgical strategy, after the ablative time, a reconstructive time is also often necessary, in a way similar to that of the surgery of cervicofacial malignant tumors, regarding mandibular reconstruction, skin graft, local flaps or free flaps. Covering the resulting defects after the ablative time with myocutaneous flaps is also useful as a healthy tissue transfer, able to create a normal revascularization [55].

**Figure 14:**
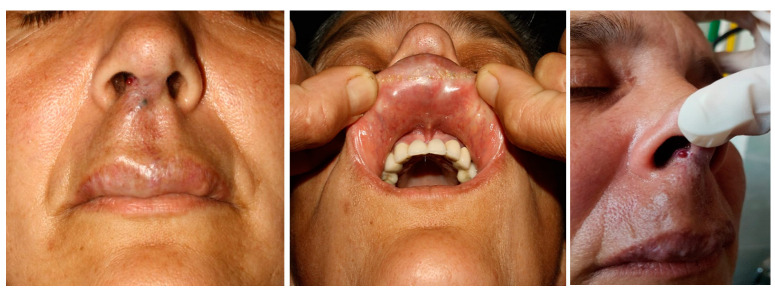
Clinical aspect of an AVM at the level of the superior lip with extension at the level of the right nasal vestibule associating with frequent epistaxis.

**Figure 15:**
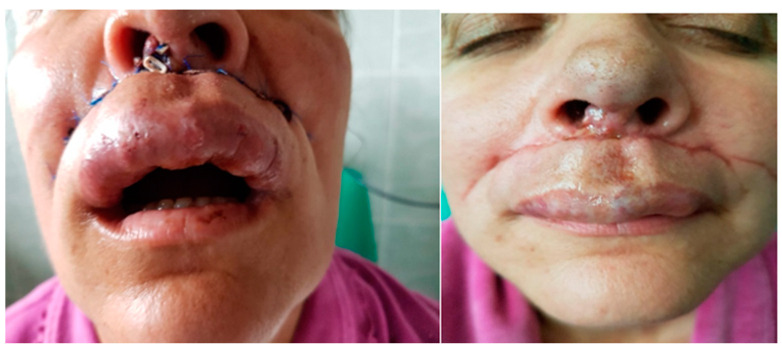
Post surgery aspect of the AVM of the superior lips undergoing selective embolization and suturing with a great aesthetic and functional outcome.

In our experience, it is much more difficult to ablatively treat and reconstruct an upper lip AVM, given the existing limitations in the current moment in this field. Therefore, in AVMs located at this level, with supply mainly from the facial artery, alternative surgical solutions to ablation can be used, such as transfixing ligation on a Dacron prosthesis for the purpose of thrombosis and reduction in size of the malformation. (Figure 14 and Figure 15). The risk of thromboembolic accidents and the partially limited aesthetic result are worth mentioning.

Synthesizing the therapeutic options in cervicofacial AVMs, we retain, as the method of choice, preoperative embolization followed by surgical ablation of the malformation in the first 48 h to prevent the development of collateral circulation, and primary embolization in particular situations when the surgery is mutilating or in case of bleeding. Other therapeutic options, such as sclerotherapy, have proven ineffective in AVMs, leading not only to the recurrence of these types of malformations, but also to their restoration to larger sizes than before treatment, representing an irritating thorn for the opening of new aberrant vessels [56].

The diagnostic and therapeutic management of AVM requires a multidisciplinary and interdisciplinary approach, involving ENT specialist, imagist, vascular surgeon, plastic surgeon, maxillo-facial surgeon, etc.

### 3.4. Combined Vascular Malformations

This group of vascular anomalies comprises several combinations of slow-flow and fast-flow vascular anomalies associated with skin changes, orthopedic problems, and aesthetic disturbances. Once the diagnosis is established, these patients require long-term multidisciplinary clinical monitoring [57].

Klippel–Trenaunay syndrome is a combination of vascular malformations with tissue overgrowth of the affected extremity. The “port wine stain” represented by capillary vascular malformations is very characteristic. Patients may develop recurrent thrombophlebitis with secondary venous insufficiency that may cause ulceration and bleeding [58]. One of the newer promising treatment strategies regarding port wine stain lesions is the use of photodynamic therapy with hemoporfin, also called hematoporphyrin monomethyl ether [59].

Gorham–Stout syndrome is a progressive lymphatic malformation with bone involvement, also known as “evaporating bone disease” due to the replacement of bone tissue with microcystic lymphatic tissue [60].

Glomuvenous malformations with an autosomal dominant genetic substrate are also described in the literature. Affected patients present with multifocal cutaneous and subcutaneous venous keratosis. These patients should be monitored regularly because these vascular abnormalities may be associated with an increased incidence of breast and thyroid cancer [61].

Imaging investigations for these combined malformations range from ultrasound with color Doppler to CT and MRI and angiography. The diagnostic and therapeutic approach to all aspects of pathology is necessary [62,63].

**Figure 16:**
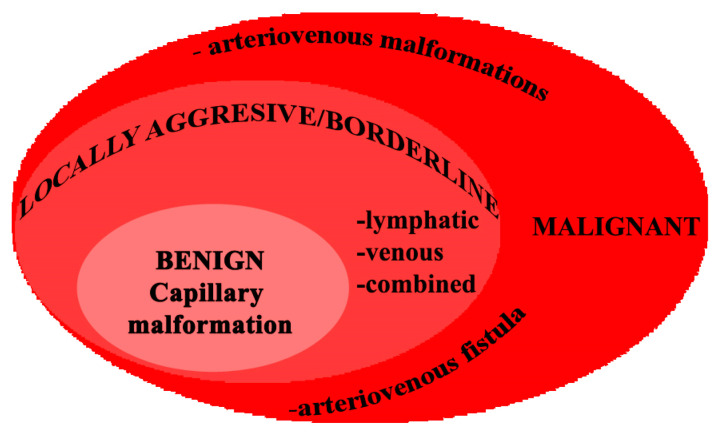
Proposed classification of cervicofacial vascular anomalies in three categories of prognosis.

In Figure 16, we propose a graphical classification of cervicofacial vascular anomalies, taking into consideration their prognosis under current treatment.

## 4. Conclusions

Cervicofacial congenital vascular anomalies represent a rare chapter of pathology with diagnostic and treatment difficulties, requiring a multidisciplinary and interdisciplinary approach. In order to have common references, a consensus of head and neck specialists regarding diagnostic and therapeutic terminology and management is important. There are two broad entities of vascular abnormalities: vascular tumors and vascular malformations. Vascular malformations are in turn divided into slow-flow malformations (venous, lymphatic, capillary or combinations) and fast-flow malformations (arteriovenous malformations and arteriovenous fistulas). Vascular tumors such as hemangiomas are characterized by the spontaneous tendency to involute once with patient age, while vascular malformations increase in size with patient age.

The anamnesis and the clinical examination guide us very well in the diagnosis. MRI imaging is essential for definitive diagnosis. Exploratory angiography is not routinely performed in vascular tumors such as hemangiomas; instead, it is mandatory as a preoperative exploration, associated with selective embolization in the case of arteriovenous malformations to minimize intraoperative bleeding. Radical ablation of the malformation ensures prevention of recurrences. Considering the diversity of clinical manifestations in the case of cervicofacial vascular anomalies, the therapeutic protocol must be individualized. The therapeutic strategy must be formulated sequentially and carried out in a mixed multidisciplinary team, trained in such pathology, in which the interventional radiologist must work in tandem with the surgeons, the anesthesiologist and the histologist.

## Data Availability

Available on request from the corresponding authors.
